# Supplementary material for: Prevalence and correlates of Helicobacter pylori infection among under-five children, adolescent and non-pregnant women in Nepal: Further analysis of Nepal national micronutrient status survey 2016
Source: PLoS Negl Trop Dis. 2021 Jun 21;15(6):e0009510. doi: 10.1371/journal.pntd.0009510 (PMC8263064; doi:10.1371/journal.pntd.0009510)
Supplement: Attachment — Submitted filename: response to reviewers HP.docx [file pntd.0009510.s001.docx]

1. We note that the following authors have not yet confirmed their authorship on this manuscript: Kedar Parajuli, Uday Narayan Yadav, Ranju Mehta, Priya Jham, Neha Mehta, Meghnath Dhimal, Dipendra Raman Singh

   At this point this request is not urgent; we have re-sent their co-author confirmation email containing an auto-confirmation link for them to click, and we will reach out to them at a later stage if we don't hear from them. We wanted to mention this in case you are aware of them experiencing technical issues with confirming authorship via the link. If so, please do ask them to contact us directly via email and we'd be happy to assist.

Response: all the authors were informed.

2. Please ensure that the Author List in your manuscript file matches the Author List in the online submission form, including initials and order. Please use either initials or full names in both manuscript and online submission form.

Response: reviewed and revised.

3. We do not publish any copyright or trademark symbols that usually accompany proprietary names, eg (R), (C), or TM  (e.g. next to drug or reagent names). Therefore please remove all instances of trademark/copyright symbols throughout the text, including "HemoCue®" on page 6.

Response: reviewed and revised.

4. Your manuscript is missing the following sections: Introduction. Please ensure these are present, and in the correct order, and that any references to subheadings in your main text are correct. An outline of the required sections can be consulted in our submission guidelines here: <https://journals.plos.org/plosntds/s/submission-guidelines#loc-parts-of-a-submission>

Response: reviewed and revised.

5. Thank you for including your ethics statement: The ethical approval to conduct the survey was approved by the Ethical Review Board (ERB) of the Nepal Health Research Council (NHRC). Please include:
i) The name(s) of the Institutional Review Board(s) or Ethics Committee(s)
ii) A statement that formal consent was obtained from the parent/guardian (must state whether verbal/written) OR the reason consent was not obtained (e.g. anonymity)

Response: reviewed and revised.
