# Supplementary material for: Prevalence and correlates of Helicobacter pylori infection among under-five children, adolescent and non-pregnant women in Nepal: Further analysis of Nepal national micronutrient status survey 2016
Source: PLoS Negl Trop Dis. 2021 Jun 21;15(6):e0009510. doi: 10.1371/journal.pntd.0009510 (PMC8263064; doi:10.1371/journal.pntd.0009510)
Supplement: Attachment — Submitted filename: Response to reviewers comments.docx [file pntd.0009510.s002.docx]

Reviewer #1: The lack of specific hypotheses makes the study way to exploratory and unfocused, despite the substantial data available regarding the burden of H pylori and the role of age, sex and poverty-related determinants
What rapid test kit was used, brand, model, date, etc. What was its Se and Sp, and impacts on results? Same for the stool antigen. Given that this is the main outcome of the study, it needs to be described in detail in the methods. And the impact of the differences between the two tests on the conclusion needs to be discussed also. How many samples were tested per plate and with repetitions or tittering levels?

Response: The methodology of H. polori testing was added.

“Stool samples were collected from non-pregnant women of 20-49 years and children of 6-59 months and were stored in a cold box in below 10 degree Celsius, and later transferred to pathologists. Then the pathologist transferred one gram of stool to a second cryovial and stored it in cold box, later transferred to Siddhi Polyclinic Laboratory in Kathmandu to test for *H. pylori*. All the frozen stool samples were stored in Walter Reed/AFRIMS Research Unit Nepal (WARUN) until lab test were performed for the detection of *H. pylori*. All the collected stool samples were examined for *H. pylori* antigen detection using an enzyme-linked immunoassay (ELISA) test kit on a Mago clinical analyzer which provides both negative and positive controls in each analytical test. For the validity of each analytical test, positive and negative controls were used where the absorbance was at least 0.8 OD units and less than 0.09 OD units, respectively. Internal quality control of the laboratory was strictly followed.”

Rapid test: “we have used the QuickVue Rapid Rest Kit for checking the H.pylori in adolescent boys and girls. The QuickVue H. pylori test detects H. pylori-specific IgG antibodies produced by individuals colonized or infected with the organism. Approximately 50µL of whole blood was added to the test cassette from the Purple Top Vacutainer. Set the timer for 5 minutes and then read the test. The result come as positive, negative or invalid.”

The sensitivity and specificity reported with biopsy was 90% and 78% respectively^1^.

What explains higher prevalence in adults when virtually no increasing trend is seen in younger individuals?

Response: the mythological differences has been presented as the limitations of study as “This study used different method for H. pylori examination for adolescents and children aged 6-59 months as well as non-pregnant women of reproductive age limit the comparability across the age group.”

Data sources, describe age of adolescents and pregnant women, instead of reporting them in the sample collection section.

Response: Reviewed and revised as per suggestions.

“Further, 1,709 children aged 6-59 months were included in this study, with the response rate of 98.9%. Likewise, 1,025 and 1,865, adolescent boys and girls aged 10-19 years were interviewed, respectively, with a response rate of 98% each. A total of 2,144 nonpregnant women aged 20-49 years was also interviewed with a response rate of 99%.”

Stata’s name is not “Software for Statistics and Data Science”, please provide the proper reference in standard format

Response: reviewed and revised as per the suggestions.

Is age considered an effect modifier? What about the differences in tests? Prevalence in adolescents is substantially lower most likely due to the tests.

Response: we do not consider age as an effect modifier due to differences in tests and this has been presented as a limitation.

Numerical covariates cannot just be adjusted as having a continuous linear relationship with the logodss of the H pylori prevalence without first verifying that this relationship is indeed linear.

Response: we didn’t check any linear relationship since we are just assessing the maximum likelihood estimates. We will revise the analysis if you don’t agree with this response.

Reviewer #2: - health status of the participants should be evaluated and clarified.
- H. pylori infection was evaluated only by rapid test kit in adolescent boys and girls.

Response: Yes, the adolescents were evaluated only by rapid test kit and the test has sensitivity 90% and specificity 78% when compared with biopsy^[1]^.

- the definition of the wealth status needs to be clarified in the text.

Response: reviewed and revised as “The wealth status was calculated based on household assets and principal component analysis was used to calculate the wealth index and further dichotomized.”

- the definition of "risk of folate deficiency" needs to be clarified in the text.

Response: reviewed and revised in the text as “Risk of RBC folate deficiency was assessed using red blood cell folate microbiological assay(29).”

Reviewer #3: The tests for H. pylori (stool antigen test (monoclonal?) and serum/whole blood antibody test) are not named. This is needed information as it can influence the reliability of the results. It is not stated at all here what was tested in blood (likely antibodies, something like “to assess H. pylori infection antibodies against the pathogen were detected in whole blood using XX Test from YY company etc”). It is also important to know how many freeze and thaw cycles have been done to the stool samples. This might have a strong impact on the antigen test. It should be also clear whether the producer of the antigen test allows the testing of frozen stool samples. The temperatures of storage should also be stated.

Response: reviewed and revised.

“Stool samples were collected from non-pregnant women of 20-49 years and children of 6-59 months and were stored in a cold box in below 10 degree Celsius, and later transferred to pathologists. Then the pathologist transferred one gram of stool to a second cryovial and stored it in cold box, later transferred to Siddhi Polyclinic Laboratory in Kathmandu to test for *H. pylori*. All the frozen stool samples were stored in Walter Reed/AFRIMS Research Unit Nepal (WARUN) until lab test were performed for the detection of *H. pylori*. All the collected stool samples were examined for *H. pylori* antigen detection using an enzyme-linked immunoassay (ELISA) test kit on a Mago clinical analyzer which provides both negative and positive controls in each analytical test. For the validity of each analytical test, positive and negative controls were used where the absorbance was at least 0.8 OD units and less than 0.09 OD units, respectively. Internal quality control of the laboratory was strictly followed.”

Rapid test: “we have used the QuickVue Rapid Rest Kit for checking the H.pylori in adolescent boys and girls. The QuickVue H. pylori test detects H. pylori-specific IgG antibodies produced by individuals colonized or infected with the organism. Approximately 50µL of whole blood was added to the test cassette from the Purple Top Vacutainer. Set the timer for 5 minutes and then read the test. The result come as positive, negative or invalid.”

The sensitivity and specificity reported with biopsy was 90% and 78% respectively^1^.

Line 145-150: the response rate seems extremely high. Was this addressed in the cited reference? How is it explained?

Response: most of the study conducted in Nepal has been reported the high response rate, DHS also has high response rate (>95%)^[2]^.

Line 189: it should read probably IgG/IgM? It would sound better IgG and IgM.

Response: reviewed and revised.

Line 188-190: it comes the question why those aged 10-19 were tested by blood test and not stool antigen test and vice versa.

Response: this has been presented as a limitation of the study.

Line 198-190: “Chi-square test was used to measure the association between the exploratory and outcome variables” This sentence is not clear. Chi-square can detect differences between groups but not measure the association, the formulation of the sentence is for a regression analysis. What were outcome variables? If I understand right there was only 1 (being infected or not).

Response: The section has deleted.


Reviewer #1: Why does table 1 lacks p-values or some test to compare groups? Also, given the sampling approach, bivariate results are probably not reasonable estimates. Bivariate tables can be integrated with regression tables, and avoid double description of the same results. There is too much redundancy in the results, the way the tables are presented
Too much emphasis on comparing boys with girls despite of not being a leading study hypothesis and no remarkable sex-related differences.
Cannot just say prevalence in differences parts of the paper due to using two very different tests.

Response: reviewed and revised as per the suggestions. Bivariate tables were integrated with the regression tables. Redundancies has been removed as much as possible.

Reviewer #2: Results need to be reconsidered (see below).

Response: reviewed and revised as per suggestions of reviewer 1.

Reviewer #3: Generally a lot of overly detailed results with minimal relevant information. Probably most tables could be provided as supplementary material. All results of an age group should be provided as one block. As there are no confidence intervals in the descriptive data tables it is very hard to see which differences are at all relevant.

Response: reviewed and revised as per suggestions of reviewer 1. Redundancies were removed, bi-variate results were integrated with the regression tables.

Line 215-216: probably “highest” and “lowest” is meant here.

Response: reviewed and revised.


Reviewer #1: Findings confirm that poverty is the main factor associated with the disease, as know from the literature. What is the novelty?

Response: this study re-conform the socio-economic is the main factors correlated with the occurrence of H. pylori infection. Also, this study did not find correlation by serum ferritin or risk of folate deficiency and *H. pylori* infection.

In the discussion, comparison of results need to take into account what tests were used for each study in each country. Even within this study, the changes in prevalence by age suggests to be test-dependent

Response: reviewed and revised as per suggestion and age related change in rates has presented as the limitation.

Reviewer #2: Conclusion is clear.

Reviewer #3: Some very important points are not addressed at all in the discussion. There were no adult men and no persons older than 49 years in study, important points as they limit the statements that can be made about the prevalence of H. pylori infection in Nepal.

Response: The limitation has reviewed and revised as per the suggestions “no adult men and no persons older than 49 years has been included in this study”

Line 389: the sentence is formulated confusingly. In the other study children above 10 were highly infected? How high? This point should be addressed as the data of the study is also from Nepal and puts into question whether methodological problems may be the cause (e.g., how was H. pylori infection detected in the other study?, If it was stool antigen testing maybe it was from fresh samples?)

Response: the line removed from the discussion due to difference in the tests used.


Reviewer #1: The English writing requires some minor adjustments and typographical mistakes
Somewhat lengthy introduction with an excessive emphasis on developed countries in the introduction, which are not relevant for comparison to the settings of Nepal. I suggest focusing on more comparable settings to the study population.

Response: the manuscript has been reviewed by a native English speaker and acknowledge in the review support received.

Reviewer #2: Editorial modification is required (see below).

Reviewer #3: Line 91-92: what is meant here by marginally? Is it then not relevant?

Response: reviewed and revised as “Gastric acidity caused by *H. pylori* can impaired iron and vitamin B12 absorption and leads to anemia”.

Line 92-93: “H pylori is cause of gastrointestinal blood loss, reduction in iron absorption and increase in iron absorption by bacteria.” This sentence does not have a reference. It is also too general with stating H. pylori is a cause of GI blood loss. Is it a relevant cause?

Response: Delated from the background section.

Line 95-97: these are more associations between H. pylori and the diseases mentioned then causing a clear decrease.

Response: reviewed and revised as per suggestions. “*H. pylori* infection associated as a protective factors for the diseases such as asthma, osteoporosis, inflammatory bowel disease, and esophageal cancer”

Line 101-104: Probably here the study from Hooi et al (Global Prevalence of Helicobacter pylori Infection: Systematic Review and Meta-Analysis) is mentioned. It would be better to cite the original study (the meta-analysis) then papers citing it. It also looks strange to cite 2 papers (14 and 15) for the same data.

Response: reviewed and revised as per suggestions.

Line 110-112: the sentence is not clear? Bloating and vomiting are risk factors for H. pylori infection? In what time period, compared to whom? Most person have bloating or vomiting sometime in their lives. Also family history of gastric cancer is probably not a risk factor for H. pylori infection but rather an association with (most likely the risk factor for cancer is H. pylori infection.

Response: since the background was lengthy I was removed the lines.

Line 113-117: In the first sentence it is not clear what is meant by longer period. The second sentence is repeating the first (acquired at an early age is during childhood). In developed countries time of infection for most persons is childhood. The whole sentence is rather confusing and too long, it should read probably something like “…acquired during childhood after which the risk of infection rapidly declines…”.

Response: reviewed and revised as per the suggestions.

Some sentences in the manuscript are very long and not formulated clearly. For example in line 81-85 it reads as if the lymphoma was colonizing the stomach: “Helicobacter pylori (H. pylori), a gram-negative and spiral-shaped bacterium previously known as Campylobacter pyloridis then C. pylori, was first isolated and identified by Warren and Marshall in 1982 (1), has been associated with gastric diseases such as peptic ulcer, chronic gastritis, gastric cancer, mucosa associated lymphoid tissue (MALT) lymphoma (2, 3) which colonizes in the gastric milieu of more than half of the global population.”

Italicization and abbreviations are not consistent in the text.

Response: reviewed and revised as per the suggestions. “*Helicobacter pylori (H. pylori),* a gram-negative and spiral-shaped bacterium previously known as *Campylobacter pyloridis* and later then *C. pylori*, was first isolated and identified by Warren and Marshall in 1982 (1). It has been associated with gastric diseases such as peptic ulcer, chronic gastritis, gastric cancer, and mucosa associated lymphoid tissue (MALT) lymphoma (2, 3) which colonizes the gastric milieu of more than half of the global population.”

Reviewer #1: This is an interesting effort, but unfocused and without clear hypotheses relevant to the global community in a topic where substantial evidence already exists. The manuscript needs a clear direction and a central topic.

Response: the objective of study has reviewed and revised as “The aim of this study was to assess prevalence and correlates (socio-demographic, nutritional status, and micronutrient status) of *H. pylori* infection in Nepal among under five children, adolescents aged 10-19 years and non-pregnant women aged 20-49 years using nationally representative data”

Reviewer #2: In this manuscript, Mehata et al investigated the prevalence of H. pylori infection among the Nepalese population. They found that poverty is associated with high prevalence of H. pylori infection.

1) In the Abstract section (l.39), mucosa associated lymphoid tissue lymphomas are indolent in nature and are rarely a cause of death, so this sentence needs to be corrected.

Response: the lines were deleted.

2) In the Background section, the authors wrote that gastric acidity caused by H. pylori leads to reduced vitamin B12 (l.91-92), but this is not true. Hypochlorhydria which occurs as a result of atrophic gastritis caused by H. pylori infection leads to reduced vitamin B12. This misleading sentence needs to be corrected.

Response: the lines were deleted as per the suggestions of other reviewers.


3) In the Background section, the reviewer could not understand the sentence "reduction in iron absorption and increase in iron absorption by bacteria" (l.93). This sentence is confusing and needs to be edited.
Response: the lines were deleted as per the suggestions of other reviewers.

4) The paragraph in the Background section from line 98 to123 has conflicting sentences within itself and is not so important for the present study. The authors should consider deleting or carefully re-editing this paragraph.

Response: the lines were deleted as per the suggestions of other reviewers.

5) Although the authors hypothesized that supply of micronutrients determines the prevalence of H. pylori infection in Nepal (l.127-128), there is no sentence in the Results or Discussion verifying this hypothesis. Furthermore, the only micronutrient assessed in this study, other than iron, is folate (described as "risk of folate deficiency"), which was not associated with H. pylori infection. The authors should investigate other additional micronutrients, such as vitamin B12, selenium, etc., if they wish to prove their hypothesis. In addition, the definition of "risk of folate deficiency" needs to be clarified in the text.

Response: added in the results and discussion.

6) Were all the participants healthy? The authors are using serum ferritin as the parameter of iron status. Some diseases, such as chronic inflammatory diseases, can lead to decreased serum ferritin independent of H. pylori infection, so the health status of the participants should be evaluated and clarified.

Response: we have stated this as a limitation of this study since this a cross sectional study unable to establish causality.

7) H. pylori positive rate was 26.2% in children at the age of 4-5, and 38.57% in non-pregnant women at the age of 20-24. However, the positive rates in adolescent boys and girls were very low at 10.5%-20.1%. While the H. pylori infection was evaluated by H. pylori antigen in the stool in children under five and non-pregnant women, it was evaluated only by rapid test kit in adolescent boys and girls. This decreased positive rate is probably due to the inadequate sensitivity of the rapid test kit. Hence, it is difficult to compare the data of adolescent boys and girls with the data obtained from subjects of other ages. The authors need to explain this limitation in the text.

Response: reviewed and revised the limitation.

8) How did the authors judge the wealth status of the participants? This needs to be clarified in the text.

Response: Added in the methodology section.

9) There are a number of errors in the Discussion section (e.g. l.370 the authors describe that H. pylori infection was significantly associated with residence area in children under the age of 5, but none of the provinces or place of residence were significantly associated with H. pylori infection./ l.405 Dalits is not significantly associated with H. pylori infection./ etc.). The authors need to carefully check the manuscript and correct the errors.

Response: reviewed and revised.

10) The reference needs to be edited to match the style of this journal.

11) There are many grammatical errors in the text. These need to be corrected.

Response: reviewed and edited carefully.

Reviewer #3: The study tries to estimate the prevalence of H. pylori infected individuals in Nepal using data and samples already available from an earlier population survey.

Line 124-130: if the prevalence of infection in Nepal is already known then it should be stated more clearly what this study adds to already available data (the study aim states “study was to assess prevalence and determinants of H. pylori”). Different study population, micronutrient data?
On the same line the sentence "Hence, we hypothesized that supply of micronutrients determines the prevalence of H. pylori
infection in Nepal." is unclear as then it is not stated how this hypothesis was then tested.

Response: the hypothesis was revised as “The infection is highly associated with malnutrition, and diarrheal diseases. Therefore, we hypothesized that supply of micronutrients such as iron, vitamin B12 deficiency, socio-economic status, and nutritional status correlates with the prevalence of H. pylori infection in Nepal.” And discussion and conclusions were revised accordingly.

1. https://www.quidel.com/sites/default/files/product/documents/EF0570905EN00.pdf
2. https://www.dhsprogram.com/pubs/pdf/fr336/fr336.pdf
