# Supplementary material for: Prevalence and correlates of Helicobacter pylori infection among under-five children, adolescent and non-pregnant women in Nepal: Further analysis of Nepal national micronutrient status survey 2016
Source: PLoS Negl Trop Dis. 2021 Jun 21;15(6):e0009510. doi: 10.1371/journal.pntd.0009510 (PMC8263064; doi:10.1371/journal.pntd.0009510)
Supplement: Attachment — Submitted filename: Response to reviewers.docx [file pntd.0009510.s003.docx]

Methods:

Reviewer #1:

The hypothesis posed in the introduction are related to nutrition-related covariates, but also include socioeconomic status, preventing a focused analysis and manuscript. This reflects in the introduction, results and section, that try to address any potential factor associated to the outcome, without focus or set direction. Similarly, the micronutrient related hypotheses end up receiving little attention and are lost in the manuscript, particularly in the central messages and conclusions in the abstract and discussion.

Response: Throughout the manuscripts is reviewed and revised considering the socio-economic, nutritional and micronutrients covariates.

Was serum ferritin deficiency analyzed as a potential factor associated to h. pylori, similar to folate deficiency, or only the association of the actual values of serum ferritin were associated to h pylori? A similar approach should be used for folate and serum ferritin, and if not, it should be justified.

Response: we have reviewed and revised the variable ‘serum ferritin level’ to the ‘iron deficiency’ using the respective cut offs for different age. Now, both the variables ‘iron deficiency’ and ‘risk of folate deficiency’ has been used as a categorical variable throughout the manuscript.

The sensitivity and specificity of the QuickVue should be reported in the paper, and it’s low specificity addressed.

Response: we have reviewed and revised as per suggestions. “The reported sensitivity and specificity of QuickVue kit 90% and 76%, respectively”. The low specificity is discussed in the limitation of study.

Similarly, Se/Sp for ELISA assays used should be reported in the methods, to be able to understand how these characteristics impact the results in the different age groups.

Response: “The reported sensitivity and specificity were 96% and 83%, respectively”, updated in the manuscript.

Where underage adolescents asked for consent to participate?

Response: Parental consent was obtained for underage adolescents as per Nepal Health Research Council (NHRC)- Ethical Review Board guidelines.

Adjusting by province may result in over adjusting and take away the effect of many variables such as differences in nutritional deficits between groups.

Response: we have conducted sensitivity analysis with and without province in the multivariate model. We didn’t find any significant change between both the models so we retain the final model having provinces.

Final model with province

| PSC_Hpylori | IRR | Std. Err. | z | P>z | [95% Conf. | Interval] |
| --- | --- | --- | --- | --- | --- | --- |
|  |  |  |  |  |  |  |
| age_cat |  |  |  |  |  |  |
| 1-2 yrs | 1.156338 | 0.433433 | 0.39 | 0.698 | 0.55466 | 2.410696 |
| 2-3 yrs | 2.418619 | 0.883497 | 2.42 | 0.016 | 1.18204 | 4.948831 |
| 3-4 yrs | 2.517665 | 0.853485 | 2.72 | 0.006 | 1.295513 | 4.892761 |
| 4-5 yrs | 2.965484 | 1.04978 | 3.07 | 0.002 | 1.481736 | 5.934997 |
|  |  |  |  |  |  |  |
| PSC_SEX |  |  |  |  |  |  |
| Female | 0.955188 | 0.124319 | -0.35 | 0.725 | 0.740123 | 1.232745 |
|  |  |  |  |  |  |  |
| caste |  |  |  |  |  |  |
| terai caste | 1.365422 | 0.443886 | 0.96 | 0.338 | 0.72202 | 2.582167 |
| Dalits | 1.634278 | 0.343553 | 2.34 | 0.019 | 1.082403 | 2.467532 |
| Janajatis | 1.178787 | 0.230865 | 0.84 | 0.401 | 0.803023 | 1.730387 |
| Muslims | 2.18408 | 0.62848 | 2.71 | 0.007 | 1.242599 | 3.838895 |
|  |  |  |  |  |  |  |
| ECO_REGION |  |  |  |  |  |  |
| Mountain | 1.040151 | 0.224799 | 0.18 | 0.855 | 0.680979 | 1.588763 |
| Terai | 1.442285 | 0.278733 | 1.9 | 0.058 | 0.987529 | 2.106455 |
|  |  |  |  |  |  |  |
| PROVINCE |  |  |  |  |  |  |
| PROVINCE-2 | 0.47071 | 0.193952 | -1.83 | 0.067 | 0.209906 | 1.05556 |
| PROVINCE-3 | 1.0479 | 0.278678 | 0.18 | 0.86 | 0.622231 | 1.76477 |
| PROVINCE-4 | 0.614873 | 0.242601 | -1.23 | 0.218 | 0.283753 | 1.332388 |
| PROCINCE-5 | 0.719101 | 0.193612 | -1.22 | 0.221 | 0.424239 | 1.218903 |
| PROVINCE-6 | 1.054042 | 0.310032 | 0.18 | 0.858 | 0.592228 | 1.875976 |
| PROVINCE-7 | 0.704086 | 0.185158 | -1.33 | 0.182 | 0.420513 | 1.178886 |
|  |  |  |  |  |  |  |
| RURAL_URBAN |  |  |  |  |  |  |
| Rural | 0.671586 | 0.145065 | -1.84 | 0.065 | 0.439783 | 1.025568 |
|  |  |  |  |  |  |  |
| WEALTH_QNT |  |  |  |  |  |  |
| Second | 1.05926 | 0.177757 | 0.34 | 0.732 | 0.762361 | 1.471786 |
| Middle | 0.80857 | 0.156527 | -1.1 | 0.272 | 0.553271 | 1.181672 |
| Fourth | 0.551012 | 0.129775 | -2.53 | 0.011 | 0.347284 | 0.874254 |
| Highest | 0.710088 | 0.198693 | -1.22 | 0.221 | 0.41033 | 1.228826 |
|  |  |  |  |  |  |  |
| wasting |  |  |  |  |  |  |
| Yes | 1.024303 | 0.284066 | 0.09 | 0.931 | 0.594795 | 1.763962 |
|  |  |  |  |  |  |  |
| stunting |  |  |  |  |  |  |
| Yes | 1.060926 | 0.183151 | 0.34 | 0.732 | 0.75638 | 1.488094 |
|  |  |  |  |  |  |  |
| underweight |  |  |  |  |  |  |
| Yes | 0.734101 | 0.152801 | -1.49 | 0.138 | 0.488181 | 1.103902 |
|  |  |  |  |  |  |  |
| Iron_def |  |  |  |  |  |  |
| Yes | 1.056369 | 0.173589 | 0.33 | 0.739 | 0.765493 | 1.457774 |
|  |  |  |  |  |  |  |
| folate |  |  |  |  |  |  |
| Yes | 1.316196 | 0.213971 | 1.69 | 0.091 | 0.957068 | 1.810081 |
| _cons | 0.138387 | 0.063241 | -4.33 | 0 | 0.056508 | 0.33891 |

With without province:

| PSC_Hpylori | IRR | Std. Err. | z | P>z | [95% Conf. | Interval] |
| --- | --- | --- | --- | --- | --- | --- |
|  |  |  |  |  |  |  |
| age_cat |  |  |  |  |  |  |
| 1-2 yrs | 1.226179 | 0.47495 | 0.53 | 0.599 | 0.573916 | 2.619748 |
| 2-3 yrs | 2.553944 | 0.949344 | 2.52 | 0.012 | 1.232552 | 5.29197 |
| 3-4 yrs | 2.649155 | 0.900549 | 2.87 | 0.004 | 1.360666 | 5.157785 |
| 4-5 yrs | 3.009241 | 1.067951 | 3.1 | 0.002 | 1.500976 | 6.033096 |
|  |  |  |  |  |  |  |
| PSC_SEX |  |  |  |  |  |  |
| Female | 0.938082 | 0.122775 | -0.49 | 0.625 | 0.725833 | 1.212398 |
|  |  |  |  |  |  |  |
| caste |  |  |  |  |  |  |
| terai caste | 1.027823 | 0.244734 | 0.12 | 0.908 | 0.644525 | 1.639067 |
| Dalits | 1.579809 | 0.351817 | 2.05 | 0.04 | 1.021047 | 2.444352 |
| Janajatis | 1.276319 | 0.226832 | 1.37 | 0.17 | 0.900909 | 1.808163 |
| Muslims | 2.117461 | 0.684158 | 2.32 | 0.02 | 1.12406 | 3.98879 |
|  |  |  |  |  |  |  |
| ECO_REGION |  |  |  |  |  |  |
| Mountain | 1.133941 | 0.240549 | 0.59 | 0.553 | 0.748205 | 1.718542 |
| Terai | 1.147607 | 0.23444 | 0.67 | 0.5 | 0.768963 | 1.712701 |
|  |  |  |  |  |  |  |
| RURAL_URBAN |  |  |  |  |  |  |
| Rural | 0.627959 | 0.16418 | -1.78 | 0.075 | 0.37617 | 1.048283 |
|  |  |  |  |  |  |  |
| WEALTH_QNT |  |  |  |  |  |  |
| Second | 1.020871 | 0.163236 | 0.13 | 0.897 | 0.746217 | 1.396615 |
| Middle | 0.815403 | 0.156767 | -1.06 | 0.288 | 0.5594 | 1.188562 |
| Fourth | 0.564075 | 0.135164 | -2.39 | 0.017 | 0.352673 | 0.9022 |
| Highest | 0.746534 | 0.183825 | -1.19 | 0.235 | 0.460735 | 1.209615 |
|  |  |  |  |  |  |  |
| wasting |  |  |  |  |  |  |
| Yes | 1.093454 | 0.304513 | 0.32 | 0.748 | 0.633508 | 1.887336 |
|  |  |  |  |  |  |  |
| stunting |  |  |  |  |  |  |
| Yes | 1.057264 | 0.183459 | 0.32 | 0.748 | 0.752455 | 1.485546 |
|  |  |  |  |  |  |  |
| underweight |  |  |  |  |  |  |
| Yes | 0.725094 | 0.149982 | -1.55 | 0.12 | 0.483423 | 1.087581 |
|  |  |  |  |  |  |  |
| Iron_def |  |  |  |  |  |  |
| Yes | 0.989311 | 0.169728 | -0.06 | 0.95 | 0.706803 | 1.384735 |
|  |  |  |  |  |  |  |
| folate |  |  |  |  |  |  |
| Yes | 1.325894 | 0.216651 | 1.73 | 0.084 | 0.962548 | 1.826396 |
| _cons | 0.123284 | 0.056705 | -4.55 | 0 | 0.050049 | 0.303682 |

Using PCA to determine a wealth index is an not sufficient description to address what that index implies and how can it be interpreted, as it does not provide an standardize well known measure but instead it results in a data-specific index. Additionally, dichotomization of a data-rich summary probably reduces excessively the diversity and variance of the wealth index. Multiple, well-defined categories will probably would be a better fit for this data, particularly because it is trying to capture the diversity within a whole country.

Response: we have reviewed and revised as per suggestions “The wealth status was calculated based on household assets recommended by Demographic and Health Survey such as: type of toilets, cooking fuels, source of water and energy, type of housing, number of living rooms, ownership of household assets, etc. Principal component analysis was used to calculate the wealth index and further wealth quintile was categorized using ranked technique to translate it to the ordinal scale.”

Analysis of numerical covariates assuming linear relationships can lead to model misspecification and the shape of the actual association should be assessed before deciding on a linear or other type of fit, making sure that the model specification responds to the data

Response: All the numerical covariates are changed in to categorical variables using standard cut offs.

No rationale is presented for analyzing boys separate from girls

Response: Nepal is a country having strong son preference has led to discriminatory practices towards girls and women, with disturbing effects on their health and nutritional status. Chaupadi Pratha [menstrual hut], early marriage, sex trafficking is still prevalent in the country. Thus, gender segregated analysis will helps to delves dipper on the correlates of H. pylori separately.

P values are used to define significance, but the actual values are not presented and without them is hard to assess the significance level

Response: Reviewed and revised in the table.

Conclusions

Reviewer #1:

The discussion continues to be unfocused, particularly without attention to the nutritional factors that were supposed to be the main hypothesis.

Response: Reviewed and revised as per suggestions. “In our study, we did not find any significant correlation between iron deficiency status and risk of folate deficiency and H. pylori infection across the age groups, which was in accordance with the findings of other studies (48) (49). However, some studies have reported association of H. pylori infection with iron deficiency, such that reduced serum ferritin levels were measured in H. pylori infected cases (50-52). In addition, there are reports of reversal of chronic iron deficiency anemia through eradication of H. pylori infection, without any additional anemia targeted treatments (53). Iron being essential factor for H. pylori growth, this bacterium may compete with host for iron uptake creating iron deficiency in hosts (54). However, from analysis of different available literature, it can be concluded that there is no universal agreement on correlation of H. pylori infection with iron deficiency. Nonetheless, the discrepancy seen in the results from different studies may also be due to different sample size or different age group of patients used in different studies, as the correlation may be age specific.”

No attention is given to the broader age differences and trends between the three study groups, the role of tests used and the difference that age implies to the epidemiology of h pylori, as age trends seem to be present in children and adolescents, but not in adults.

Response: The age differences H. pylori positive rate was revised as in the limitation section: “26.2% in children at the age of 4-5 years, and 38.6% in non-pregnant women at the age of 20-24 years. However, the positive rates in adolescent boys and girls were very low at 10.5%-20.1%. While the H. pylori infection was evaluated by H. pylori antigen in the stool in children under five and non-pregnant women, it was evaluated only by rapid test kit in adolescent boys and girls. This decreased positive rate is probably due to the inadequate sensitivity of the rapid test kit. Hence, it is difficult to compare the data of adolescent boys and girls with the data obtained from subjects of other ages.”

The limitations of the study are only listed and not really discussed or addressed as they may have important challenges.

Response: Reviewed and revised as per suggestions: “This study is the largest nationwide epidemiological study to provide a comprehensive understanding of *H. Pylori* prevalence and correlated potential risk factors in Nepal. The study used systematic random sampling to estimate the prevalence, which enabled the recruitment of participants from diverse demographic backgrounds. This study has a few limitations. Because this study is cross-sectional, causality cannot be inferred from the findings. We were unable to account for several confounding factors, such as household sanitation status, family history and dietary habits, and thus residual confounding could not be eliminated. Future research should account for these factors. Furthermore, serology may create bias in differentiating past and present infection as antibody titers may remain high even after treatment. *H. pylori* positive rate was 26.2% in children at the age of 4-5 years, and 38.6% in non-pregnant women at the age of 20-24 years. However, the positive rates in adolescent boys and girls were very low at 10.5%-20.1%. While the *H. pylori* infection was evaluated by *H. pylori* antigen in the stool in children under five and non-pregnant women, it was evaluated only by rapid test kit in adolescent boys and girls. The differences in positive rate is probably due to the inadequate sensitivity and specificity of the both the test kits, the reported positive and negative predictive value of rapid test kit were 81% and 88%, respectively (31). The positive and negative predictive values of ELISA test kit were 96% and 91% respectively (32). Hence, it is difficult to compare the data of adolescent boys and girls with the data obtained from subjects of other ages. Moreover, this study did not evaluate the health status of participants, such as chronic inflammatory diseases, can lead to decreased serum ferritin independent of H. pylori infection, and may have impacted the results. Also, this study did not account for prior treatment with antibiotics or proton pump inhibitors. This study also did not assess the correlation of infection rate with symptoms and disease process. This study did not include adult men and no persons older than 49 years.”

Editorial and Data Presentation Modifications?

Reviewer #1:

English writing, only in the abstract: under five children, adolescent and … / We studied prevalence, / data from Nepal Micronutrient Survey / while blood sample was used / No hypothesis listed in abstract.

Response: Reviewed and revised as per suggestions “*Helicobacter pylori* infection is a global health problem, with most of the cases occurring in developing countries, widely known as ‘a poor man’s pathogen’. The risk factors for H. pylori infections are poverty, overcrowding, and unhygienic conditions, which are common problems in under-privileged countries such as Nepal. Despite having a high risk of H. pylori infections, there are no national level study has been conducted to assess prevalence and correlates of H. pylori infection in Nepal. Therefore, we hypothesized that supply of micronutrients such as iron, vitamin B12 deficiency, socio-economic status, and nutritional status correlates with the prevalence of H. pylori infection in Nepal.”

Review comments are submitted with the understanding that the authors will respond to each comment by making changes in the actual manuscript to address each comment. Responses in the letter are useful, but actual changes in the manuscript are necessary, as readers will probably have similar concerns as those of reviewers.

Response: All the necessary changes have been made carefully in the manuscript.

Summary and General Comments:

Reviewer #1:

The changes made in this revised version of the manuscript do not feel that are properly addressing the concerns of this reviewer, as changes feel mainly superficial. A proper assessment of the role of nutritional deficiencies in H pylori prevalence would require more in-depth attention and focus.

Response: We have reviewed and revised the manuscript based on our outmost knowledge considering the reviewers comments.
